# Measuring Persistence in the US Equity Gender Diversity Index

**DOI:** 10.1007/s11205-023-03104-x

**Published:** 2023-04-05

**Authors:** Juan Infante, Marta del Rio, Luis A. Gil-Alana

**Affiliations:** 1 University Villanueva, 28034 Madrid, Spain; 2 University of Navarra, NCID and DATAI, Pamplona, Spain; 3 Universidad Francisco de Vitoria, Madrid, Spain

**Keywords:** Gender diversity index, Persistence, Fractional integration, C22, G15, M21

## Abstract

An analysis of the SPDR SSGA Gender Diversity Index ETF using fractional integration or I(d) techniques and daily data from 8 March 2016 to 8 January 2021, reveals that the series is highly persistent with an order of integration smaller than, though very close to 1. However, when estimating d recursively across subsamples, two peaks can be observed. The first peak appears in the sample with 679 observations (ending at 26 December 2018) and the second one occurs in the sample with 974 observations and ending at 28 February 2020, which shows the most significant change in d, moving from values within the I(1) interval to values significantly above 1. The findings indicate that the Covid-19 pandemic has had a significant impact on the persistence of the SPDR SSGA Gender Diversity Index ETF, increasing its magnitude and thus the level of persistence.

## Introduction

The aim of the ETF SPDR SSGA Gender Diversity Index is to provide investment results that mirror the total return of an index that tracks U.S. firms which have demonstrated a commitment to gender diversity in their boards and governing bodies as a way to promote the growth of women through diversity.

This index is intended to evaluate the success of large U.S. firms considered as “*gender diverse*”, that prioritize gender diversity in their upper-level management positions. This index is constructed using the Index Universe, which ranks companies in each sector according to the degree of gender diversity according to different criteria based on ratios, as determined by an analysis conducted by an independent third party based on information contained in the company's regulatory filings, press releases and corporate website ("*company communications*"). This ranking classifies each sector based on three criteria related to the gender diversity ratio: (i) the ratio of men to women in executive roles and on the board of directors, (ii) the ratio of men to women in executive roles compared to all executive roles, and (iii) the ratio of female executives (excluding those on the board) to all executives (excluding those on the board).

This paper aims to use fractional integration methods to analyze the statistical properties of the ETF SPDR SSGA Gender Diversity Index, examining the degree of persistence of the series. These methods are more general than standard econometric models, which are solely based on I(0) stationary and I(1) non-stationary cases, and therefore only utilize integer degrees of differentiation. This makes fractional integration an appropriate tool for this analysis. Furthermore, integration order can help to distinguish between temporary and permanent disturbances in the series, which is highly relevant from an economic policy point of view and enables more dynamic model specifications.

The objective of the paper is twofold. First, we investigate the degree of persistence of the SPDR SSGA Gender Diverstiy Index in order to determine if shocks in the series have permanent or transitory effects. Then, once the order of integration of the series has been established, we examine if it has been constant across the sample period, or if, alternatively, has changed due to the shocks occurred in the series in the time period examined. Our results indicate that the series is highly persistent, with an order of integration that is close to 1, and due to the sanitary crisis created by the Covid-19 pandemic, this order of integration has substantially increased in the most recent period.

The remainder of this paper is organized as follows: Sect. 2 reviews the literature of the SPDR SSGA Gender Diversity Index ETF. Section 3 presents the data and results. Finally, Sect. 4 offers a conclusion.

## A Short Review of the Literature

For an investment to be considered “*socially responsible*”, it must comply with Environmental, Social and Governance (ESG) criteria. A sustainable product and behaviour is considered to reduce inefficiency, improve the use of resources and imply an innovation that means a cost reduction in the long term (Clark et al., 2015).

The company's Corporate Social Responsibility (CSR), in the same vein, has a direct impact on its systematic risk. Alburquerque et al. (2014) conducted a study in which it was shown that CSR measures reduce a company's systematic risk (also known as beta) by increasing consumer loyalty. In the case of those companies that have a high CSR score, this beta is reduced even in the case of separately analyzing the components of community, diversity, employee relations, environmental and human attributes or aspects.

The United Nations has listed Gender Equality as the fifth Sustainable Goal. Companies have found that gender diversity creates better organizations with greater creativity, flexibility and innovation and better teamwork.

The principle of Diversity is the result of the changes that culture undergoes through time and space, and that characterizes social groups. This diversity must not only be recognized, but every effort must also be made to strengthen it, since it constitutes a factor of development in areas such as economic, intellectual, affective, moral and spiritual (Montoro and Lopez-Herrerías, 2008).

The diversity criterion tries to unify the principle of “*equality*”, used since the time of the illustration, with the recognition of the uniqueness of the human person on which subjectivity is based. The balance between equality and the valuation of individual and intergroup singularity represents the two conceptual pillars on which the development of the diversity strategy is based (Barberá & Ramos, 2004).

Gender diversity, on the contrary, is part of the potential values that women have, not of their rights. These values contribute to the organization and acquire a special strength in times of radical social and labour change such as the one we are experiencing at the moment. Women represent a fundamental added value within society. Is the society, and not only women, who must demand rights for themselves. It is no longer a question of women joining the social organization on equal terms, but a complete social restructuring in which all people have a place and in which women’s work skills are positively valued.

According to Alburquerque et al. (2014), gender diversity has a positive impact on financial and business performance, in addition to improving human capital management at all organizational levels of a company and permitting a framework of equal opportunities for employees.

Fang et al. (2018) suggested that companies with female leaders tend to be more profitable, have better employee retention rates, and demonstrate higher levels of morale. Corporate governance is not entirely effective without true managerial diversity (Gaurav & Seema, 2016). Having women on the board of directors gives more importance to CSR policies and ensures greater sensitivity to them (Williams, 2003) and is an indication of more participatory decision-making (Konrad et al., 2008). But, on many occasions, the presence of a female manager may not be enough since the rest of the board may consider her as to be just a token and it is difficult for women to raise their voices on any subject and make their opinion heard. (Brewer & Kramer, 1985; Kanter, 1977; Lord & Saenz, 1985; Nemeth, 1986; etc.).

Although, at present, an organization's relationship with its stakeholders is continually evolving as the focus shifts from the need of the shareholder to the needs of various stakeholders the society. In this regard, Zahra et al. (1989) explain that boards of directors have focused their efforts on effective management to protect shareholder interests, because it is evident that board composition is an important factor with a direct impact on performance and Corporate Social Responsibility initiatives. The diversity of the board and its relationship to CSR is one of the areas that is growing rapidly and that brings great value to a company.

## Data and Results

The SPDR SSGA Gender Diversity Index ETF utilizes a sampling strategy to track the performance of the SSGA Gender Diversity Index (the "*Index*"). This means that the Fund is not obligated to purchase all of the securities found in the Index. To create a portfolio of securities that closely matches the Index in terms of risk and return, the Fund may purchase a subset of the Index's securities. The Fund's holdings are determined by a number of factors, most notably the size of the Fund's assets. Analyzing these factors, the Fund's investment adviser, SSGA Funds Management, Inc. ("*SSGA FM*" or the "*Adviser*"), following what it deems most appropriate in order to meet the Fund's objective, may invest the Fund's assets in a subset of the securities in the Index, or may invest the Fund's assets in all of the securities represented in the Index in approximately the same proportions as the Index.

On the annual Index rebalance determination date, the components of the index are initially weighted based on the market capitalization of the free float. Subsequently, so that the maximum weighting of each security included in the Index is limited to 5%, an adjustment is made to the component weights so that the aggregate sector weights of the index are equal to the aggregate sector weights of the Index Universe. The Index is reset annually on July 15 or the first business day after that if July 15 falls on a non-business day. Total annual Fund operating expenses is 0.2%. The Net Asset Value is the price provided for any single day of data series. It is calculated as the market value of the SPDR SSGA Gender Diversity Index ETF total assets, minus liabilities, divided by the number of outstanding shares. Market Value–Determined by the midpoint between the bid/offer prices as at the closing time of the New York Stock Exchange (typically 4:00PM EST) on business days.

State Street Global Advisors (the "*Index Provider*" or "*SSGA*"), an affiliate of the Fund and its Advisor, SSGA FM, created and sponsors the Index. The Index Provider is in charge of establishing and upholding the rules for creating and calculating the weights of the securities in the Index.

**Fig. 1:**
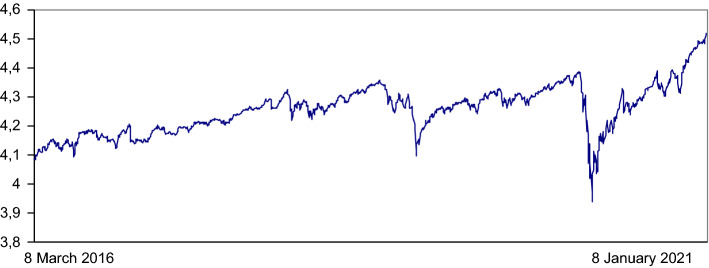
Time series: SPDR SSGA Gender Diversity Index ETF

We report in Table 1 some descriptive statistics on the original data, while Fig. 1 displays the time series plot in logarithm form. It can be observed that the values seem to increase across time though we also observe two important drops, one at December 26, 2018 and the second one at February 28, 2020. The drop in values in December, 26 2018 was due to an interest rate hike announced by the United States Federal Reserve, and fears of a partial government shutdown due to the budget disagreement between Republicans and Democrats. Another factor that helped to further precipitate the decline were statements that it would be difficult to reach a permanent economic agreement between Washington and Beijing in the tariff war. On the other hand, on February 28, 2020 stock markets around the world reported their biggest declines in a week since the 2008 financial crisis, as the uncontrolled expansion of Covid-19 caused fear in the financial market.

**Table 1:** Daily data SPDR SSGA gender diversity index ETF

Starting date	March 8, 2016
Ending date	January 8, 2021
Frequency	Daily
No of observations	1192
Mean	70.58
Maximum value	91.63
Minimum value	51.20
Standard dev	5.84

The methodology used is based on fractional integration, that is, allowing for fractional degrees of differentiation in the original data to get an I(0) behaviour. The estimated model is the following:

1 $$ x_{t} \,\,\, = \,\,\,\,\alpha \,\,\, + \,\,\,\,\beta \,t\,\,\,\, + \,\,\,\,z_{t} \,,\,\,\,\,\,\,\,\,\,(1\,\, - \,\,L)^{d} z_{t} \,\,\,\, = \,\,\,\,u_{t} ,\,\,\,\,\,\,\,\,\,\,\,t\,\,\,\, = \,\,\,\,1\,,\,\,2\,,\,\,...\,, $$

where x_t_ is the time series we observe (in logs), α and β are unknown coefficients referring respectively to a constant and a linear time trend, L is the lag-opertor, i.e., Lz_t_ = z_t-1_, and the regression errors, z_t_ are I(d), so that u_t_ is an I(0) process that will be assumed first to be uncorrelated (white noise) and later, weakly autocorrelated. The parameters are estimated using the Whittle function in the frequency domain (Dahlhaus, 1989) by using a simple version of the testing approach developed in Robinson (1994) widely used in numerous empirical applications.

We first conducted standard unit root methods. In particular, we tried with ADF tests (Dickey & Fuller, 1979) along with some others, namely Phillips and Perron (1988), Elliot et al. (1996) and Ng and Perron (2001), and the results, thougn not reported, supported the unit root hypothesis in all cases. Nevertheless, it must be pointed out that most of these procedures have very low power in the context of fractionally integrated alternatives (see, e.g., Diebold & Rudebusch, 1991; Hassler & Wolters, 1994; Lee & Schmidt, 1996; etc.). In Table 2 we display the estimates of d (and its 95% confidence bands) for the three classical cases of no deterministic terms (i.e., α = β = 0 in (1)), an intercept (β = 0), and an intercept with a linear time trend, assuming that the error term u_t_ in (1) is first a white noise process and then autocorrelated by following the exponential spectral model of Bloomfield (1973). We have marked in bold in the table, the selected model for these deterministic terms, based on the significance of the t-values of these coefficients in the d-differenced regressions. Note that inserting the second equality in (1) into the first one, by means of multiplying by (1-L)^d^ each term in the first equality produces a regression model where the errors are u_t_, and thus, I(0) by construction, implying that the t-values of the estimated coefficients hold. Thus, if both coefficients (the constant and the time trend) are statistically significant, we choose that model and report the estimates of d in the last column of the tables; if, on the contrary, the time trend coefficient is insignificant, we choose the model with only an intercept (the estimates of d reported in column 3); finally, if both coefficients are insignificant, we choose then the model with no constant and no trend (column 2).

**Table 2:** Estimated values of d

	No terms	An intercept	A linear time trend
White noise errors	1.00 (0.96, 1.04)	**0.93 (0.89, 0.97)**	0.93 (0.89, 0.97)
Autocorrelation	1.00 (0.94, 1.08)	**1.12 (1.04, 1.22)**	1.12 (1.04, 1.22)

Bold values indicate estimation of d (and its 95% confidence bands) for classical case of no deterministic terms with an intercept (β = 0)

The first thing we observe in Table 1 is that the time trend is unrequired in the two cases of white noise and autocorrelated errors, the intercept being sufficient to describe the deterministic terms. Focusing on the differencing parameter, the estimated value of d is equal to 0.93 under white noise errors, and 1.12 using the model of Bloomfield (1973); in the former case the unit root null hypothesis (i.e., d = 1) cannot be rejected and this hypothesis is rejected in favour of d > 1 under autocorrelation. Nevertheless, the hypothesis of mean reversion (d < 1) is statistically rejected in both cases, implying permanency of shocks.

**Fig. 2:**
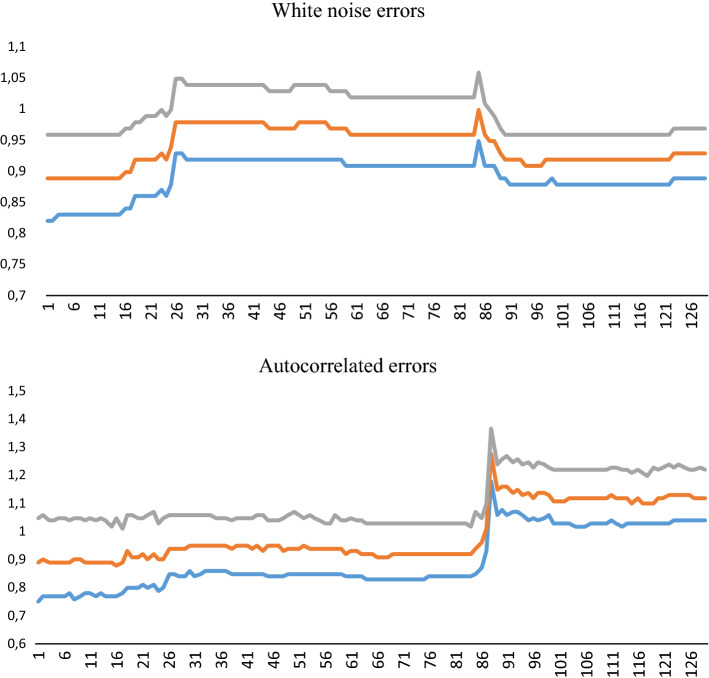
Recursive estimates of d

Next, we questioned whether the differentiation parameter has remained constant over the entire sample period by first re-estimating d for a subsample ending at observation 554 (June 28, 2018**)**, and then, re-estimating d in successive sub-samples adding five observations each time until the last observation in the sample. The results based on white noise errors are reported in the upper part of Fig. 2, while the lower part refers to the case of autocorrelated disturbances. In both cases we observe a similar pattern with two jumps changing the level of d at December 26, 2018 and at February 28, 2020. Under the white noise specification, we observe that evidence of mean reversion (i.e., significant evidence of d < 1) is found before the first break and after the second one, but in the period between both breaks there is a substantial increase in d moving away from mean reversion. With autocorrelation, which seems to be more realistic since a more flexible structure is permitted, the most significant break seems to be the second one, showing an increase in d from the I(1) case to I(d) with d > 1. This second specification seems to be more realistic based on the potential structure on the error term. Thus, the results seem to support the hypothesis of “*no mean reversion*”, with the shocks caused by the sanitary crisis permanently affecting the series and requiring strong measures by the practitioners, policy makers and authorities if the aim is to recover the original trends in the data.

## Conclusions

In this paper, we have investigated the persistence of the EFT SPDR SSGA Gender Diversity Index by employing fractional integration or I(d) techniques. Data collected from March 8, 2016 to January 8, 2021 was gathered on a daily basis and has been taken into consideration. As a result it has been obtained that this series is highly persistent and has a lower order of integration, very close to 1. Anyway, by estimating d recursively through subsamples an interesting result is obtained. The first peak in the estimation of d starts around the sample with 679 observations (and ending at 26 December 2018), while the second one occurs around the 974^th^ observation and ending at 28 February 2020, being this latter shock the one producing the strongest change in persistence. In fact, it produces a change in the estimation of the differencing parameter from values within the I(1) interval to values significantly above 1. We also observe that if the errors are uncorrelated, evidence of mean reversion (i.e., significant evidence of d < 1) is found before the first break and after the second one, but in the period between both breaks there is a substantial increase in d moving away from mean reversion. It seems the drop in values in December, 26 2018 due to an interest rate hike announced by the United States Federal Reserve which may have been provoked by fears of a partial government shutdown due to the budget disagreement between Republicans and Democrats and the difficulty of reaching a permanent economic agreement between Washington and Beijing in the tariff war. This is also observed in the model with autocorrelation. Covid-19’s sanitary crisis on February 28, 2020, on the other hand, has had an evident effect in the degree of persistence in the SPDR SSGA Gender Diversity Index ETF, increasing its value, and this is even more evident in the model with autocorrelated errors. Further research must be done in the series in order to determine the duration of the present sanitary shock.

## Data Availability

The corresponding author can provide the datasets generated and/or analysed during this study upon reasonable request.
